# RSpred, a set of Hidden Markov Models to detect and classify the RIFIN and STEVOR proteins of *Plasmodium falciparum*

**DOI:** 10.1186/1471-2164-12-119

**Published:** 2011-02-18

**Authors:** Nicolas Joannin, Yvonne Kallberg, Mats Wahlgren, Bengt Persson

**Affiliations:** 1 Department of Microbiology, Cell and Tumor biology (MTC), Karolinska Institutet, SE-17177 Stockholm, Sweden; 2 Department of Cell and Molecular Biology (CMB), Karolinska Institutet, SE-17177 Stockholm, Sweden; 3 IFM Bioinformatics and Swedish e-Science Research Centre (SeRC), Linköping University, SE-58183 Linköping, Sweden

## Abstract

**Background:**

Many parasites use multicopy protein families to avoid their host's immune system through a strategy called antigenic variation. RIFIN and STEVOR proteins are variable surface antigens uniquely found in the malaria parasites *Plasmodium falciparum *and *P. reichenowi*. Although these two protein families are different, they have more similarity to each other than to any other proteins described to date. As a result, they have been grouped together in one Pfam domain. However, a recent study has described the sub-division of the RIFIN protein family into several functionally distinct groups. These sub-groups require phylogenetic analysis to sort out, which is not practical for large-scale projects, such as the sequencing of patient isolates and meta-genomic analysis.

**Results:**

We have manually curated the *rif *and *stevor *gene repertoires of two *Plasmodium falciparum *genomes, isolates DD2 and HB3. We have identified 25% of mis-annotated and ~30 missing *rif *and *stevor *genes. Using these data sets, as well as sequences from the well curated reference genome (isolate 3D7) and field isolate data from Uniprot, we have developed a tool named RSpred. The tool, based on a set of hidden Markov models and an evaluation program, automatically identifies STEVOR and RIFIN sequences as well as the sub-groups: A-RIFIN, B-RIFIN, B1-RIFIN and B2-RIFIN. In addition to these groups, we distinguish a small subset of STEVOR proteins that we named STEVOR-like, as they either differ remarkably from typical STEVOR proteins or are too fragmented to reach a high enough score. When compared to Pfam and TIGRFAMs, RSpred proves to be a more robust and more sensitive method. We have applied RSpred to the proteomes of several *P. falciparum *strains, *P. reichenowi, P. vivax*, *P. knowlesi *and the rodent malaria species. All groups were found in the *P. falciparum *strains, and also in the *P. reichenowi *parasite, whereas none were predicted in the other species.

**Conclusions:**

We have generated a tool for the sorting of RIFIN and STEVOR proteins, large antigenic variant protein groups, into homogeneous sub-families. Assigning functions to such protein families requires their subdivision into meaningful groups such as we have shown for the RIFIN protein family. RSpred removes the need for complicated and time consuming phylogenetic analysis methods. It will benefit both research groups sequencing whole genomes as well as others working with field isolates. RSpred is freely accessible via http://www.ifm.liu.se/bioinfo/.

## Background

Many pathogens have evolved strategies to survive within the hosts they infect. One strategy consists of varying the antigens the pathogen exposes to its host immune system, usually resulting in the proliferation of multicopy protein families, commonly named Variable Surface Antigens (VSA) [1]. In the case of the malaria parasite *Plasmodium falciparum*, there are three major VSA that allow the parasite to avoid the host's immune system and establish chronic infections: the *Plasmodium falciparum *Erythrocyte Membrane Protein 1, RIFIN and STEVOR proteins (reviewed in [2,3]).

The RIFIN and STEVOR families are groups of VSA proteins that are unique to the *Plasmodium falciparum *and *P. reichenowi *parasites [4-9]. They are only present in two species, but they number more than 200 copies per genome. Although the genome of *Plasmodium falciparum *has been fully sequenced [6], the information obtained for the reference strain does not represent the full knowledge of these antigenic variant protein families. Field isolates investigated for their repertoire of *rif *and *stevor *genes show an extensive variability [10,11]. This hypervariability makes these proteins difficult to study and their primary function(s) remain to be discovered. A recent analysis of the whole *rif *gene repertoire, which encode for RIFIN proteins, from the reference genome has concluded that this family can be sub-divided into functionally distinct groups [12]. One of these sub-groups, A-RIFIN, as well as the STEVOR proteins are predominantly exposed to the host's immune system at the surface of the infected red blood cell (RBC) [4,7,8].

Sequestration of infected RBCs is a virulence factor that allows the parasite to avoid passage through the spleen, therefore increasing its chances of survival. A recent analysis of gene expression of VSA of a *P. falciparum *strain isolated from a splenectomized patient showed that *A-rif *and *stevor *genes were not expressed [13], whereas, in isolates from normal patients, these genes are expressed [4,7,10,11]. The authors relate this loss of expression to the loss of the sequestration phenotype. Conversely, *B-rif *genes are expressed regardless of the absence of this virulent phenotype [13]. These differences in phenotype as well as in the localization of these proteins [4,11,14,15] and the predicted sub-functionalization of RIFIN proteins [12] demonstrate the importance of distinguishing each of these sub-groups. 

**Figure 1:**
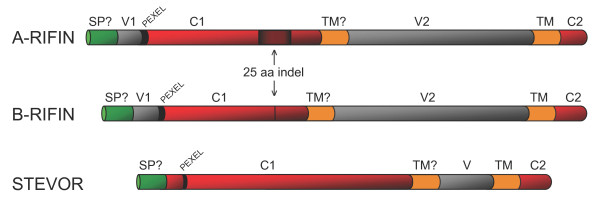
**Schematic representation of RIFIN and STEVOR proteins**. Schematic representation of A-RIFIN, B-RIFIN and STEVOR proteins (not to scale). Aa: amino acid; Indel: insertion/deletion; SP?: potential signal peptide; V1: first variable region; PEXEL: *Plasmodium *export element; C1: first conserved region; TM?: questionable transmembrane region; V2: second variable region; TM: highly probable transmembrane region; C2: second conserved region.

Figure 1 shows a schematic representation of A-RIFIN, B-RIFIN and STEVOR proteins, including the potential signal peptide (SP?), variable regions (V1 and V2),*Plasmodium *export element motif (PEXEL) [16,17], conserved regions (C1 and C2) and finally the two predicted transmembrane regions, first a questionable one (TM?) and second a highly probable one (TM). 

Currently, the RIFIN and STEVOR protein families are represented by the Pfam domain PF02009 [18]. However, this hidden Markov model (HMM) fails to distinguish RIFIN from STEVOR proteins. There are TIGRFAMS HMMs [19] that do separate RIFIN and STEVOR proteins, but they fail to classify the RIFIN or STEVOR proteins into sub-groups. Although STEVOR, A-RIFIN and the different B-RIFIN groups are identifiable by experts, they require cumbersome phylogenetic methods to be divided into their respective sub-groups [12]. In this study we report the development of a tool, consisting of a set of HMMs and an evaluation program, to automatically sort RIFIN and STEVOR proteins according to their sub-groups. We have named the tool RSpred for RIFIN and STEVOR predictor.

## Results

### Curation of the RIFIN and STEVOR repertoires of the *Plasmodium falciparum *DD2 and HB3 genomes

We have carried out manual curation of the RIFIN and STEVOR repertoires in the DD2 and HB3 draft genomes. We used BLAST to detect the DD2 and HB3 sequences, using the entire 3D7 *rif *and *stevor *gene repertoire as query and the DD2 and HB3 supercontigs as databases. This allowed us to detect all potential *rif *and *stevor *genes.

**Figure 2:**
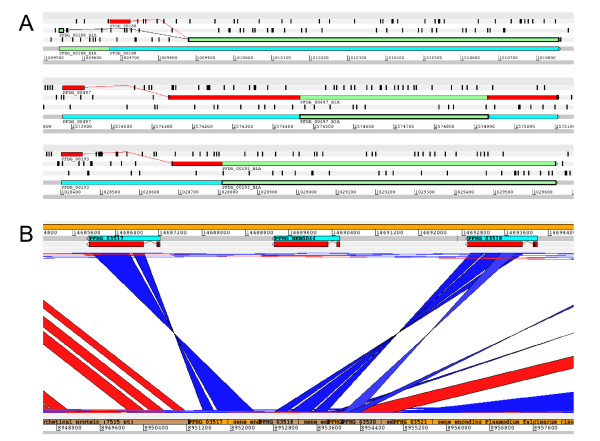
**Missing genes and corrections from automatic predictions**. (A) Artemis visualization of Broad Institute Annotations (BIA) and manual annotations. Manually curated genes are represented in blue, proteins in red. BIA genes and proteins are in green. (B) Artemis Comparison Tool visualization of the BLAST results between HB3 supercontig vs. HB3 automatically predicted genes. A missing gene, labelled "New gene" in this figure, is visible between two predicted genes. Blue and red represent BLAST hits between the (upper) supercontig and the (lower) predicted genes. BLAST hits are coloured according to the strand on which the hit is found (red for the Crick strand, blue for the Watson strand).

We compared these BLAST hits with the automatically generated annotations provided by the Broad Institute. Although most of our manually curated genes correspond to automatic annotations, we have revised the exon-intron boundaries for more than 25% of them (three examples shown in Figure 2A). In addition to these modifications, we have found some odd predictions: four of our manually curated genes had automatic predictions as two genes, interrupted by a frame shift or stop codon, and one had been predicted as a shorter hypothetical gene on the opposite strand (data not shown). Finally, we have detected 30 genes that had no automatic predictions at all (example shown in Figure 2B). The naming system of the DD2 and HB3 predicted genes uses the format PFDG_XXXXX and PFHG_XXXXX, where XXXXX is a number. Currently, there are 5380 and 5623 predicted genes for DD2 and HB3, respectively. We have decided to annotate the new genes using incremental numbering from 5381 for DD2 and 5624 for HB3, i.e. PFDG_05381 and PFHG_05624. Additionally, we have appended all the RIFIN and STEVOR genes, manually curated for this study from DD2 and HB3, with "-NJ" in order to distinguish them from the original and future annotations. All curated genes from the DD2 and HB3 draft genomes (193 and 178, respectively) are deposited in the antigenic variation database varDB [20].

### Sub-grouping, a new take on the matter

We needed curated data sets of sequences belonging to each group in order to train the HMMs. STEVOR and RIFIN proteins share little similarity, which makes them easy to distinguish from one another after completion of multiple sequence alignment with known STEVOR and RIFIN sequences. Full-length A-RIFIN and B-RIFIN proteins are easily recognized, upon visual inspection of multiple sequence alignments, based on the presence (A-RIFIN) or absence (B-RIFIN) of a fairly conserved 25 amino acid residue indel in the conserved region (Figure 1). However, the sub-groups within the B-RIFIN cluster are not so easily sorted without the help of phylogenetic analysis.

Previous research, based on the RIFIN repertoire of the reference genome, describes three sub-groups in the B-RIFIN cluster: B1-, B2- and B3-RIFIN [12]. Our present analysis confirms the integrity of the B1- and B2-RIFIN sub-groups. However, we find that there is too little coherence (less than 50% average pairwise identity in the reference strain, and low confidence bootstrap scores in the phylogenetic trees) within the B3-RIFIN cluster to make it form a defined sub-group. We propose to redefine these sequences simply as B-RIFIN.

**Figure 3:**
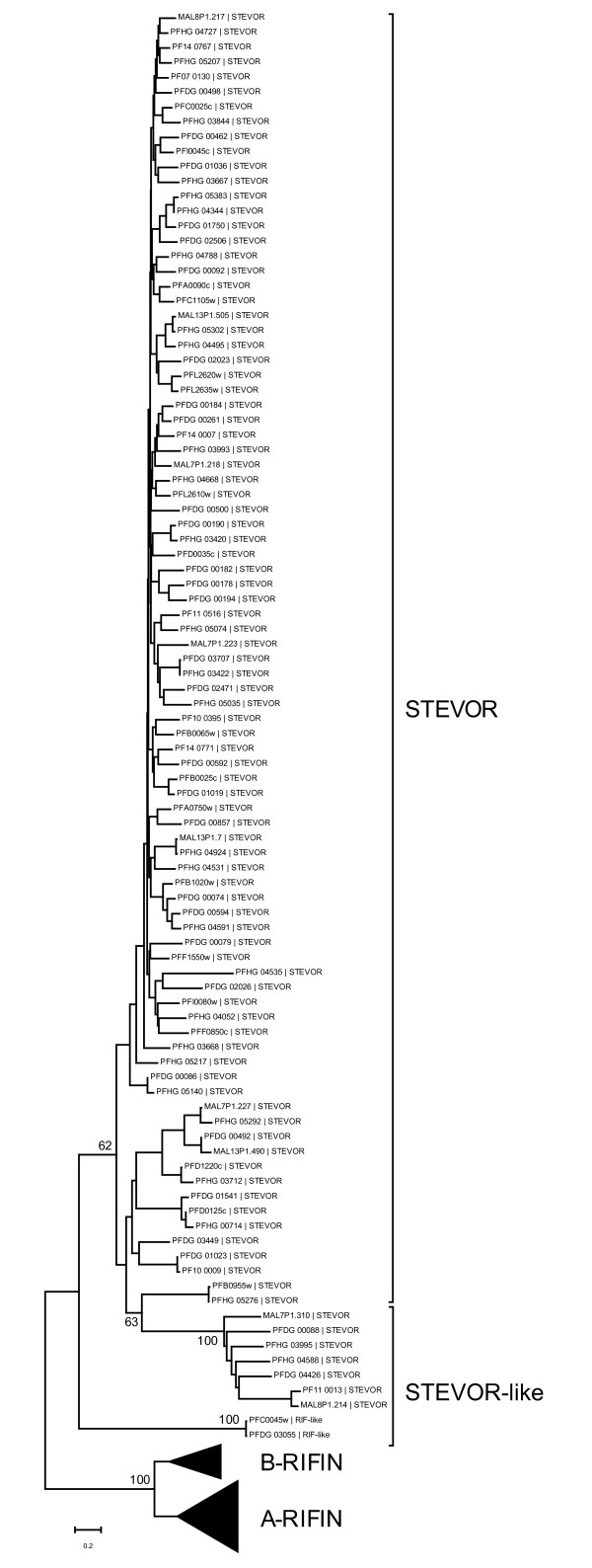
**Phylogenetic tree of RIFIN and STEVOR proteins**. The phylogenetic tree shows the segregation of low scoring STEVOR proteins, which we call STEVOR-like. RIFIN sub-groups have been collapsed to improve readability of the tree. Bootstrap support (in %), after 500 replicates, is only shown for values >60%.

We also investigated the homogeneity of the STEVOR family. In phylogenetic trees, derived from multiple sequence alignments of STEVOR proteins of sequences obtained from the three *P. falciparum *genomes, 3D7, HB3 and DD2, the majority of STEVOR proteins forms a cluster. However, a small group of proteins, which we call STEVOR-like, cluster separately from the main STEVOR group (Figure 3). These sequences differ from typical STEVOR proteins by different amino acid compositions from the signal sequence through the majority of the conserved domain. Also, the variable domain's length is less consistent than in most STEVOR proteins. Regardless of these differences, STEVOR-like proteins share short amino acid motifs throughout the protein, as well as the entirety of the very typical C-terminus, with STEVOR proteins.

### Sorting out the results and limits of detection

**Figure 4:**
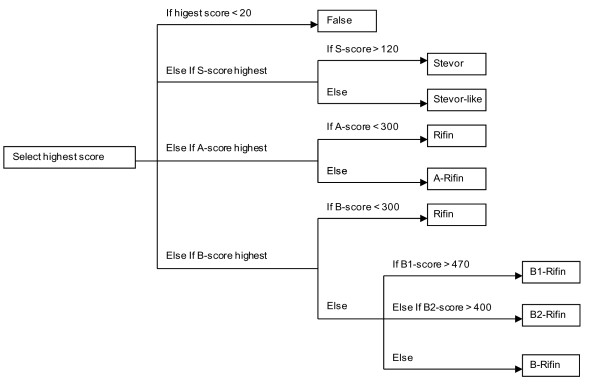
**Evaluation program**. The evaluation program compares the scores of all HMMs in database searches. Depending on each sequence's scores, the program designates the sequence as "False", i.e. neither STEVOR nor RIFIN, or as belonging to one of the groups: STEVOR, STEVOR-like, RIFIN, A-RIFIN, B-RIFIN, B1-RIFIN or B2-RIFIN.

A program was created to evaluate the results obtained when the five HMMs were used in database searches. This program uses cut-offs to determine the proper call for each sequence (Figure 4). Since there are several cut-offs, our method includes several limits of detection (LOD).

The first LOD is the detection of sequences as True or False: whether they are RIFIN or STEVOR sequences or neither. Any score <20 is considered False, i.e. not a RIFIN or a STEVOR. Of all the curated sequences in our dataset, three have scores <20: PFDG_05381, PFDG_04771 and PFDG_04350. The first protein, PFDG_05381, is an extremely short protein derived from a gene at the end of the supercontig 1.45. The sequencing coverage and assembly of contig ends are often questionable, generating erroneous sequences; therefore it is not surprising that this protein is not detected with the STEVOR HMM. The second protein, PFDG_04771, is one of the three sequences of the rifA2 group described by Wang *et al. *[21]. The two other rifA2 sequences, PFD0070c and PFHG_03700, are among the proteins with the lowest of all the positive A-RIFIN HMM scores (60.9 and 63.8 respectively). These three sequences are extremely similar to each other with the exception of a short variable region preceding the C-terminal transmembrane domain. In the case of PFDG_04771, it is a low complexity repeat of a SSGGS motifs. Additionally, this sequence is missing its N-terminal end. We assume that these circumstances, as well as the divergence of the rifA2 proteins from the basic RIFIN type, reduced its score below the detection limit. Although these sequences are full length (with the exception of PFDG_04771), all other low scoring (but higher than any rifA2) A-RIFIN sequences are fragments, again stressing the atypical properties of rifA2. The third protein below the first LOD, PFDG_04350, is a partial sequence (119 residues) covering only the C-terminal part of the protein. It is most similar to PFL2585c, a protein with very atypical N- and C-terminal ends, although the majority of the protein is typical of A-RIFIN proteins. The limited length and odd sequence of PFDG_04350 prevent its recognition as a RIFIN protein. Thus the three proteins failing to reach the first LOD have too little sequence similarity to be identified as RIFIN or STEVOR sequences.

The second LOD is specific to STEVOR proteins: if the score against the STEVOR HMM is higher than the True/False cut-off, but <120, then the sequence is reliably related to STEVOR proteins, but either differs from typical STEVOR sequences or is too fragmented to reach a high enough score. We refer to these potential STEVOR sequences as STEVOR-like proteins. The protein fragment PFHG_05644 is an example of low confidence sequence (score < 120) that we assign as STEVOR-like, although it probably is a valid STEVOR fragment. Among the sequences that score <120 with the STEVOR HMM are two identical sequences, PFC0045w and PFDG_03056, found in the 3D7 and DD2 strains, respectively. The PlasmoDB version 7.1 annotation for the PFC0045w protein is "RIFIN". However, although they are distinct from STEVOR proteins, our phylogenetic analysis clearly shows that these sequences are not RIFIN proteins, as they tend to cluster separately from the RIFIN and closer to STEVOR proteins. Until we can accumulate more sequences of this type, RSpred will predict these proteins to be similar to STEVOR and will assign them the STEVOR-like tag.

The third LOD is specific to RIFIN proteins: if the score against either the A-RIFIN or the B-RIFIN HMM is higher than the score against the STEVOR HMM, but <300, then the sequence is reliably a RIFIN protein, but it is not possible to identify its sub-group. Typical examples are fragments of proteins, e.g. PFDG_04007, PFHG_05281 and A1KQT0 (from DD2, HB3 and Uniprot respectively). In several cases, the short length of the sequence and the absence of determining properties (e.g. the 25 amino acid residues indel) result in these sequences having low scores against both the A-RIFIN and the B-RIFIN HMMs. Some rare proteins include enough of the conserved C1 region to identify them as A- or B-RIFIN, but nevertheless score <300 and are thus sorted into the RIFIN group. These sequences are most often truncated sequences or contain very odd amino acid composition, e.g. PFDG_02116 and PFHG_03477, respectively, possibly caused by low sequencing coverage or genome assembly problems.

Finally, the fourth limit of detection concerns B1- and B2-RIFIN proteins: if the score against the B-RIFIN HMM is >300, but the B1- and B2-RIFIN HMMs do not reach the cut-offs, then the protein will be evaluated as B-RIFIN instead of its proper sub-group. Among all the sequences from our curated dataset, we have not detected any false negative B1- or B2-RIFIN sequences.

### Automatic detection of RIFIN and STEVOR sub-groups in draft genomes

We applied our HMMs to all coding sequences (CDS) equal to or longer than 100 amino acids from 15 draft genomes (downloaded from the Broad Institute of Harvard and MIT [22] and the Welcome Trust Sanger Institute [23]) that do not have available annotations. The screening of these CDS gave variable results, depending on the genome, from 76 to 286 RIFIN and STEVOR sequences detected (see Table 1 for the distribution per sub-group). Although most of these genomes have been sequenced to a very low coverage (1.25×), each sub-group was detected in almost all genomes. The only exceptions are the 7G8 genome in which B1-RIFIN proteins were not found and FCC-2_hainan in which B2-RIFIN proteins were not detected. Interestingly the *Plasmodium reichenowi *genome had the highest number of hits.

**Table 1:** Prediction of RIFIN and STEVOR proteins in 15 draft genome datasets

Dataset	Size	A-RIFIN	B-RIFIN	B1-RIFIN	B2-RIFIN	RIFIN STEVOR STEVOR-like			Total
**7G8**	21756	32	13	0	2	20	9	4	80

**D10**	18305	31	9	1	2	12	15	6	76

**D6**	17468	37	19	2	2	21	15	4	100

**sDD2**	21348	84	33	4	4	29	30	7	191

**FCC-2_hainan**	20080	44	15	2	0	26	14	5	106

**HB3**	21641	94	21	7	1	17	31	6	177

**IGH-CR14**	20321	107	39	6	5	12	33	9	211

**IT-strain**	20215	82	29	5	3	20	33	7	179

**K1**	16559	42	15	5	3	25	16	6	112

**P_reichenowi**	6957	124	45	11	10	60	32	4	286

**RAJ116**	17252	45	18	4	2	22	3	2	96

**RO-33**	20632	59	14	3	4	28	20	7	135

**Santa_Lucia (SL)**	16889	43	8	2	1	24	10	4	92

**Senegal_v34.04**	15786	59	10	1	1	30	17	4	122

**VS_1**	24738	71	34	5	3	39	25	10	187

All CDS, equal to or larger than 100 amino acids in length, from publically available draft genomes were scored with RSPred.

### Negative datasets

Currently, RIFIN and STEVOR proteins have only been found in *Plasmodium falciparum *and the related *P. reichenowi*. Neither Pfam nor TIGRFAMs detect these proteins in any other known species. Additionally, orthology prediction tools and databases do not yield any RIFIN or STEVOR homologues in any other species [24-26]. Finally, the investigation of other *Plasmodium *multigene families have not detected any RIFIN or STEVOR homologous proteins [27,28]. Hence, we decided to use other *Plasmodium *species as negative controls. No RIFIN or STEVOR sequences were predicted in *P. vivax*, *P. yoelii*, *P. berghei*, *P. knowlesi *or *P. chabaudi*. RSpred was also run against the entire Uniprot database, but there were no RIFIN or STEVOR sequences predicted, except for those belonging to *P. falciparum*.

### Comparison with Pfam and TIGRFAMs

Other prediction methods exist for the RIFIN and STEVOR protein families, although each one has its limitations. Pfam [18] only predicts if a sequence is a RIFIN/STEVOR (PF02009) or not, while TIGRFAMs [19] only separates RIFIN (TIGR01477) from STEVOR (TIGR01478) proteins. Additionally, the TIGRFAMs were trained as global models and therefore do not detect sequence fragments. None of the two predict RIFIN sub-groups, as RSpred does.

In order to test the sensitivity of the three methods, we applied them to the set of RIFIN and STEVOR sequences that were not used for the training of RSpred. Out of 339 RIFIN/STEVOR sequences, RSpred identified 338 (99.7%) of them, whereas Pfam detected 332 (97.9%) and TIGRFAMs only detected 297 (87.6%). Both TIGRFAMs and Pfam fail to identify low scoring STEVOR, and the former also fails to identify fragments. The sorting of RIFIN and STEVOR proteins into sub-groups makes RSpred more specific than the other models. In addition, RSpred detects more sequences than Pfam and TIGRFAMs; it is therefore also the most sensitive of the three methods.

## Discussion

### Redefining the RIFIN and STEVOR sub-groups

Previous studies describe RIFIN and STEVOR sequences as a large group of related proteins unique to *P. falciparum*. Subsequent analysis of the RIFIN protein family, based on the reference genome, showed that the RIFIN family can be further sub-grouped into A- and B-RIFIN sequences and the latter divided into B1-, B2- and B3-RIFIN [12].

Our current analysis, which includes many more sequences, confirms the sub-division of RIFIN sequences into A-, B1- and B2-RIFIN groups, which all have defined characteristics. However, it is an overstatement to create a defined group for the remaining B-RIFIN sequences. These sequences represent a heterogeneous cluster (10 genes in the 3D7 reference strain) of sequences that are defined by the fact that they are not A-RIFIN sequences and have relatively little similarity to B1- and B2-RIFIN proteins. We have therefore decided to retrograde the B3-RIFIN sequences to the rank of B-RIFIN.

A recent study has defined potential sub-groups within the A-RIFIN sequences, rifA1 and rifA3. These groupings rely on sequence similarity of 71% and 84% and, for a large majority, their genomic location in a head-to-head orientation with group A var genes [21]. We have not trained HMMs to recognize these groups because of the low number of sequences available from the curated datasets. Also, we find that there are several other such sub-group candidates, but the small number of sequences within a single genome makes it difficult to distinguish between *bona fide *sub-groups and recently expanded genes.

These authors also defined a sub-group, rifA2, which is composed of one divergent RIFIN sequence that is present, with 78% conservation, in all genomes investigated [21]. The case of single copy genes that are very conserved between genomes are possibly better classified as conserved genes rather than sub-groups. Also, we have noted that the proteins that compose the rifA2 group score the lowest of all RIFIN sequences, with one of them predicted as "false". The fact that partial A-RIFIN protein sequences score higher than the full length rifA2 and the divergence of these sequences from typical RIFIN proteins strongly suggests that these are related to RIFIN proteins but have a different function not requiring multiple copies for the survival of the parasite.

In this study, we have only focused on the three genomes (3D7, HB3 and DD2) for which annotations are available as well as the Uniprot database that contains data from field studies. We confirm the finding, by Wang *et al. *[21], that several RIFIN sequences are relatively conserved across strains, however it is difficult to evaluate whether this represents a measure of the divergence of parasite populations or if they have been evolutionarily selected for specific functions.

Also, we have chosen to adopt a conservative approach to the STEVOR designation. All sequences that are clearly related to STEVOR sequences, but that do not score high enough will be tagged STEVOR-like by the RSpred program.

### Ambiguous sequences

Four sequences predicted to be A-RIFIN proteins also had relatively high scores (> 300) with either the B1- or the B2-RIFIN HMM. Upon closer inspection of these sequences, applying phylogenetic analysis to alignments of each half of these proteins, it appears that their N-terminal half correspond well with A-RIFIN sequences whereas their C-terminal half is characteristic of B1- or B2-RIFIN proteins (data not shown). These sequences are hybrids between A- and B1/2-RIFIN proteins and confirm previous reports of recombination as a mean for the diversification of these VSA gene families [29].

### Advantages, limits and utility of RSpred

We have named our set of HMMs and the evaluation program RSpred, for RIFIN and STEVOR predictor. We have shown that it efficiently detects RIFIN and STEVOR proteins and classifies them according to their sub-group. Although there are no false positive detections, RSpred is conservative with truncated and remotely related sequences. However, most of these sequences are at least recognized and predicted as RIFIN or STEVOR proteins. Finally, RSpred proves to be more sensitive than the existing Pfam and TIGRFAMs HMMs [18,19], which are also limited in the scope of their classification, as they do not recognize RIFIN or STEVOR sub-groups.

We have applied RSpred to whole proteomes extracted from novel genome assemblies. Although these genomes are mostly sequenced to a very low coverage (1.25×), we were able to detect all sub-groups within these genomes. This resource will be increasingly useful as more genomes are being sequenced: in particular, there is a large *Plasmodium *genome sequencing project [30] that is scheduled to sequence over 100 *Plasmodium *parasite genomes, which will allow for meta-genomic analysis of the RIFIN and STEVOR protein families.

## Conclusions

The analysis of proteins that are members of large families is often overwhelming due to the difficulty to assign proper classification. The RIFIN and STEVOR families are such groups of proteins: complications are in part due to their large diversity within each parasite's genome, but even more so with the extreme diversity between parasite populations [4,5,10,11,31]. Our prediction tool, RSpred, is designed to simplify the classification of these proteins into previously identified sub-groups [6,12] with the following benefits:

• It eliminates the need to manually retrieve reference sequences and perform multiple sequence alignments;

• It eliminates the need for any prior knowledge of these protein families in order to sort them properly;

• It out performs existing tools;

• It identifies and sorts RIFIN proteins into RIFIN, A-RIFIN, B-RIFIN, B1-RIFIN and B2-RIFIN.

Although these groups probably have diverged in function [12], the sequence conservation between these proteins assumes that their respective functions are still closely related. Greater knowledge of the smaller sub-groups B1- and B2-RIFIN proteins will improve our understanding of the larger A-RIFIN and STEVOR groups that play a more preponderant role at the surface of the infected host cell [4,13].

## Methods

### Data sets, retrieval and curation

We obtained sequence information from several sources, including PlasmoDB [32], Uniprot [33], the Welcome Trust Sanger Institute [23] and the Broad Institute of Harvard and MIT [22].

#### 3D7 sequences

We used search functionalities of the PlasmoDB v6.3 to retrieve all proteins annotated as RIFIN and STEVOR (221 sequences) excluding MAL7P1.208 that is annotated as RIFIN-like but is more similar to Rhoptry Associated Membrane Antigen (RAMA) proteins.

#### DD2 & HB3 retrieval and curation

We downloaded all data files pertaining to the DD2 and HB3 genomes (version 1) from the Broad Institute website [22].

The Supercontigs of both DD2 and HB3 were searched against the 3D7 repertoire of *rif *and *stevor *genes using BLASTn [34]. The BLAST results were visualized using Artemis and ACT (Artemis Comparison Tool) [35,36]. Each hit in the draft genomes was manually checked for the presence of a Broad Institute annotation (BIA). Generally, three case scenarios would occur:

1. Either there was an annotated gene corresponding to the manually curated *rif *or *stevor *gene. In this case, the gene would take the BIA gene name.

2. Or there was an annotated gene that did not quite overlap with the manual curation. In this case, the manually curated gene would take the BIA gene name.

3. Or there was no annotated gene at or near those coordinates. In this case, a new gene would be annotated with a new name.

We detected 193 and 179 RIFIN and STEVOR sequences from DD2 and HB3, respectively.

#### Field isolate data

We retrieved all RIFIN and STEVOR protein sequences from the Uniprot Knowledgebase [33] (446 sequences). We then removed all sequences from the 3D7 reference genome (215 sequences after filtering).

#### Additional draft genomes

Finally, we retrieved additional draft genome sequences from the Broad Institute and Welcome Trust Sanger Institute websites [22,23]. The additional genomes downloaded from the Broad Institute were *Plasmodium falciparum *supercontigs files of 7G8 nucleus, D10 nucleus, D6 nucleus, Fcc-2/Hainan nucleus, RO-33 nucleus, Santa Lucia (SL) nucleus, K1 nucleus, Senegal_V34.04 nucleus, VS/1 nucleus, IGH-CR14 nucleus, RAJ116 nucleus http://www.broadinstitute.org/annotation/genome/plasmodium_falciparum_spp/MultiDownloads.html and from the Welcome Trust Sanger Institute were the *Plasmodium falciparum *Ghanaian Isolate contigs version 20080302 ftp://ftp.sanger.ac.uk/pub/pathogens/Plasmodium/falciparum/Ghanaian_Isolate/ and IT strain supercontigs version 2007114.phusion ftp://ftp.sanger.ac.uk/pub/pathogens/Plasmodium/falciparum/IT_strain/Archive/, as well as the *Plasmodium reichenowi *contigs version 031104 ftp://ftp.sanger.ac.uk/pub/pathogens/Plasmodium/reichenowi/.

These sequence data were produced by the Broad Institute and Welcome Trust Sanger Institute, respectively.

At the time of writing, these genomes have no official annotations; therefore, using Artemis, we extracted from them all coding sequences (CDS) equal to or greater than 100 amino acids long, regardless of the presence of a start codon (see Table 1).

### Sequence analysis for sub-group determination

All alignments were carried out using MAFFT or Kalign 2, with default parameters [37,38]. We used Jalview and Bioedit for alignment visualization and editing [39,40]. Phylogenetic analysis was carried out with Molecular Evolutionary Genetic Analysis 4 (MEGA 4) [41]. All phylogenetic trees were built with the Neighbor-Joining method, considering gaps and missing data as pairwise deletions and using the Amino: Poisson correction model. Phylogenetic trees were tested with 500 bootstrap replicates.

We first aligned all sequences together in order to distinguish STEVOR and RIFIN proteins from each other. During this process, we detected a small subset of sequences that are related to STEVOR proteins but do not have a high enough HMM score. These sequences will be tagged as STEVOR-like until the availability of more sequences will allow for better categorization.

The RIFIN sequences were subsequently sub-divided according to the classification described in Joannin *et al. *[12]. A first approximation of the sub-grouping relies on the presence or absence of the characteristic 25 amino acid sequence that is present in A-RIFIN but absent in B-RIFIN proteins [6,12,42]. Sequences, which were either truncated or contained large indels, that were not identifiable as A- or B-RIFIN according to this criterion, were gathered into an "Unknown RIFIN" group. The remaining RIFIN sequences (A- and B-RIFIN) were aligned and sorted into groups according to the resulting phylogenetic tree. Sequences were grouped into A-RIFIN, B-RIFIN, B1-RIFIN, B2-RIFIN, modified from Joannin *et al. *[12] with the B3-RIFIN sub-group here renamed as B-RIFIN (see Results), as well as an "Ambiguous" subgroup. The Ambiguous group gathered all sequences that were identifiable as A-or B-RIFIN sequences but were not resolved in the phylogenetic trees.

### HMM training, testing and evaluation program

The HMMs for the five different groups of RIFIN and STEVOR sequences were built using HMMER2 [43]. Both global and local build options were tried and the local (hmmbuild-f) was found to perform best with this type of data, containing full length as well as truncated and fragmented sequences.

For the purpose of HMM training, all alignments were created using Mafft-linsi [37]. A number of protein sequences were either truncated compared to typical sequences or contained indels. We decided that sequences should be complete and typical from the PEXEL motif (Plasmodium Export Element motif) [16,17] to the C-terminal transmembrane domain; the alignments were constrained to start at this motif as well. The five training sets were made non-redundant using FASTA [44], so that the final sets contained no sequence with more than 80% identity to any other. Outliers were removed using a jack-knifing test. During this test each sequence in the training set was excluded, one at a time, an alignment created and a new HMM built. The removed sequence was scored against this new HMM, together with every sequence from the other training sets (i.e. a negative dataset). If the excluded sequence did not score higher than every sequence from the negative dataset it was removed from the final training set. The final training sets consisted of 259 A-RIFIN, 96 B-RIFIN, 26 B1-RIFIN, 9 B2-RIFIN and 51 STEVOR sequences.

A program, written in C, was created to manage the results obtained when the five HMMs were used in database searches. Figure 4 displays the decision process and the cut-offs. The cut-offs were set using the manually curated dataset as 'truth', including the odd sequences (with respect to the amino acid composition or sequence length) removed from the final training set.

### Control data sets

In order to test our HMMs for false positives, we retrieved the proteomes of several other *Plasmodium *species. All plasmodium specific datasets where downloaded from PlasmoDB version 7.1[32] and downloaded protein coding sequences from *Plasmdium falciparum *3D7 (5418, version: 2010-06-01) [6], *Plasmodium vivax *Sal-1 (5393, version: 2007-06-13) [45], *Plasmodium chabaudi chabaudi *(5123, version: 2010-06-01), *P. knowlesi *strain H (5194, version: 2010-06-01) [46], *P. yoelii yoelii *strain 17XNL (7724, version: 2005-09-01) [47] and *P. berghei *strain ANKA (4857, version: 2010-06-01) [48]. Additionally, we used the original Broad Institute annotated protein sequences from the DD2 (5380, version: 2007-04-13) and HB3 (5623, version: 2007-03-16) genomes [22].

## Authors' contributions

NJ participated in the conception and design of the study; he performed the data collection and curation, the phylogenetic analysis and analyzed all results; he drafted and revised the manuscript. YK participated in the design of the study; she trained the HMMs and made the evaluation program as well as analyzed all results; she revised the manuscript. MW revised the manuscript. BP participated in the design of the study and revision of the manuscript. All the authors have read and approved of the final manuscript.
